# Visual spatial cue use for guiding orientation in two-to-three-year-old children

**DOI:** 10.3389/fpsyg.2013.00904

**Published:** 2013-12-02

**Authors:** Danielle van den Brink, Gabriele Janzen

**Affiliations:** ^1^Behavioural Science Institute, Radboud University NijmegenNijmegen, Netherlands; ^2^Donders Institute for Brain, Cognition and Behaviour, Radboud University NijmegenNijmegen, Netherlands

**Keywords:** spatial cognition, spatial development, individual differences, frames of reference, optic flow, spatial updating, landmarks, spatial exploration

## Abstract

In spatial development representations of the environment and the use of spatial cues change over time. To date, the influence of individual differences in skills relevant for orientation and navigation has not received much attention. The current study investigated orientation abilities on the basis of visual spatial cues in 2–3-year-old children, and assessed factors that possibly influence spatial task performance. Thirty-month and 35-month-olds performed an on-screen Virtual Reality (VR) orientation task searching for an animated target in the presence of visual self-movement cues and landmark information. Results show that, in contrast to 30-month-old children, 35-month-olds were successful in using visual spatial cues for maintaining orientation. Neither age group benefited from landmarks present in the environment, suggesting that successful task performance relied on the use of optic flow cues, rather than object-to-object relations. Analysis of individual differences revealed that 2-year-olds who were relatively more independent in comparison to their peers, as measured by the daily living skills scale of the parental questionnaire Vineland-Screener were most successful at the orientation task. These results support previous findings indicating that the use of various spatial cues gradually improves during early childhood. Our data show that a developmental transition in spatial cue use can be witnessed within a relatively short period of 5 months only. Furthermore, this study indicates that rather than chronological age, individual differences may play a role in successful use of visual cues for spatial updating in an orientation task. Future studies are necessary to assess the exact nature of these individual differences.

## Introduction

Spatial orientation refers to the ability to maintain body orientation in relation to the surrounding environment at rest and during motion, and is considered a necessary prerequisite for successful navigation. We rely on spatial orientation when we navigate through familiar environments in our daily lives, when we plan an optimal route from our current location to our home, or even when we guide an avatar through a virtual world in a video game. Positioning of a self within an environment and representation of the position of objects and environmental features with respect to each other, as well as a continuous update of this knowledge, are considered key elements in spatial orientation (Hunt and Waller, 1999). The ability to maintain orientation is crucial also for toddlers, as it will for example, help them to relocate their parents in case they have momentarily escaped from their parent's attention in a new environment. The current study investigated orientation abilities on the basis of visual spatial cues in toddlers (2.5–3-year-olds), and assessed individual factors that possibly influence their spatial task performance.

There is general agreement that location can be represented in two fundamental ways to allow successful orientation and navigation (O'Keefe and Nadel, 1978; Gallistel, 1990; Kosslyn, 1994; Newcombe and Huttenlocher, 2000). One system involves the representation of positions of objects in relation to the observer, called *egocentric coding*. It can be used when the observer is stationary or when he/she is able to track his/her movement based on optic flow, vestibular and proprioceptive cues, a process known as path integration. The second system is independent of the observer's current position and involves an externally referenced spatial coding based on inter-object relations, referred to as *allocentric coding*. In adults there is growing evidence for a two-system model of parallel spatial-representational systems provided by body and environment in object-location memory (Simons and Wang, 1998; Wang and Simons, 1999; Mou et al., 2004; Nadel and Hardt, 2004; Burgess, 2006; Waller and Hodgson, 2006). Studies investigating the cerebral organization of spatial processing in patients and healthy adults provide evidence for specific, although partially overlapping, neural circuits mediating egocentric and allocentric representations (Ghaem et al., 1997; Maguire et al., 1998; Mellet et al., 2000; Committeri et al., 2004; Janzen and Jansen, 2010).

In the developmental literature, research questions initially focused on operant spatial coding systems in infants and children at various stages of development. For instance, the *egocentric-to-allocentric-shift theory* proposes that the body is the primary coding system available to the infant, and that allocentric spatial representations develop later in life, having a longer maturational trajectory (Piaget and Inhelder, 1948; Acredolo, 1990; Campos et al., 2000). An alternative theory claims that both coding systems are already present and operant in infants. However, rather than a genuine shift in abilities, a change in predominance of the use of a particular spatial coding system is proposed, from a predominant egocentric coding system in infants to a predominant use of allocentric representation system in adults, when both systems are in conflict (Rieser, 1979). Although relevant in terms of linking spatial coding abilities at various developmental stages to adult frames of reference use and their neural underpinnings, the description of spatial abilities in mere terms of egocentric or allocentric processing has certain disadvantages. Recently, research has focused more on assessment of spatial development as characterized by *what* spatial information is used at various stages of development rather than *how* this information is encoded in terms of spatial reference frames (Newcombe et al., 2013).

### Spatial cue use in young children

Previous studies investigating spatial cue use in infants and young children have revealed that already at a very young age infants are able to use a variety of spatial cues for (re)orientation purposes across multiple task contexts. Between 4.5 and 12 months there is evidence of rudimentary tracking of one's position (path integration) based on passive or active movement cues (Rieser and Heiman, 1982; Landau and Spelke, 1988; Schmuckler and Tsang-Tong, 2000; Kaufman and Needham, 2011), and of use of adjacent landmarks (so-called beacons) after rotation (Acredolo and Evans, 1980; Crowther et al., 2000; Lew et al., 2000). Between 12 and 18 months, children have been shown to be able to use the individual features of landmarks to locate a goal, but only if oriented (Lew et al., 2006). Between 18 and 24 months toddlers are capable of relocating hidden objects using a combination of path integration and visual featural information, such as landmarks (Newcombe et al., 1998; Newcombe and Huttenlocher, 2000), and of using the geometry of enclosed spaces (e.g., the relative length of the walls in a rectangular room) following disorientation (Hermer and Spelke, 1994, 1996; Learmonth et al., 2002). Between 3 and 5 years there is evidence of a refinement in landmark use. For instance, around this age children are capable of using distal landmarks such as buildings to reorient in an open parkland (Smith et al., 2008), and more proximal landmarks in large (but not small) testing rooms (Learmonth et al., 2008). Finally, between the age of 5 and 8 years a development of viewpoint independence in spatial memory can be witnessed. Within this time frame children develop the ability to retrieve locations from any arbitrary viewpoint, without path integration cues (Nardini et al., 2006, 2009). However, even at age 8 children fail to successfully combine certain sources of information about spatial location (i.e., self-motion and landmark cues), alternating between them in conflict situations. Conversely, adults show evidence of integrating these cues in a Bayesian fashion, weighting them close to optimally to reduce variance (Nardini et al., 2008).

To date, the influence of individual differences in skills relevant for orientation and navigation has not received much attention in spatial development research, and a detailed analysis of how individual children learn to rely on specific spatial cues and how to weight these cues in an optimal fashion is lacking. It has been proposed that individual differences in children's orientation abilities will likely be affected by their spatial action, such as motor development and the child's opportunities to explore its surroundings (Newcombe and Ratliff, 2007). For instance, the ability to crawl at 8 months has been shown to improve infants' search performance after a 180° rotation (Bai and Bertenthal, 1992). Other studies suggest that language may play a significant role in spatial cognition (Hermer-Vazquez et al., 1999; Pyers et al., 2010; Shusterman et al., 2011). For example, 5–6-year-old children who produced the spatial expressions involving “left” and “right” outperformed children who did not on a search task requiring successful landmark-based reorientation (Hermer-Vazquez et al., 2001). In the present study, we made a first attempt at linking different developing individual behavioral capacities to visual spatial cue use for orientation in toddlers.

### Orientation based on visual cues

Successful orientation after a spatial transformation can be achieved by means of spatial updating of one's own position or by means of viewpoint independent processing. Viewpoint independent processing refers to the cognitive process that computes the spatial relationship between visual cues such as objects and geometry cues to encode the topological structure of the environment, and it allows for detection of a hidden target from a novel vantage point, even when the viewpoint change is not produced by the child's own movement (Nardini et al., 2006; Bullens et al., 2010). In spatial updating the spatial relationship between an individual and his/her surroundings is computed based on perceptual information (e.g., visual, vestibular and proprioceptive cues) about the individual's own movements.

To date, many studies investigating infant or toddler spatial orientation abilities have included self motion, and/or geometry of an environment (e.g., rectangular rooms). Some studies have shown that in the presence of geometry cues children from 24 months on are capable of finding an object following disorientation (Hermer and Spelke, 1994, 1996; Lee and Spelke, 2010a), and that children aged 3.5 years are capable of using self-motion cues alone to encode the location of objects (Nardini et al., 2006). Particularly, in a study by Bremner et al. (1994), children aged 36 months had acquired the skill to use proprioceptive and visual flow information to keep track of their position in space whereas 24-month-olds were less successful.

It has been debated whether visual information alone is sufficient for spatial updating (Klatzky et al., 1998; Wang, 2004). Several studies investigating adult spatial updating have suggested that vestibular and proprioceptive information are important, and that optic flow is not sufficient (Klatzky et al., 1998). Yet some studies show that visual information provided by Virtual Reality (VR) can be adequate to trigger spatial updating processes during rotational transformations (Wraga et al., 2004; Riecke et al., 2007). In addition, a recent study by Bremner et al. (2011) using a rotating room shows that infants as young as 6–14 months old take both vestibular and optic flow information into account in real life orientation. Furthermore, a study by Schmuckler and Jewell (2007) shows that 6-month-old infants also benefit from visual information provided by simulated self-movement (based on a video recording of a moving camera in first person perspective) when watching a hidden toy reappear from a correct or incorrect container while enabled to track the correct container. These results show that physical movement is not necessarily a prerequisite for spatial updating.

Together these findings suggest that children's performance at different age levels is either highly sensitive to subtle changes in a study's design (i.e., task and spatial cues available) and/or possibly influenced by individual differences. The latter is likely to have an impact since the variability in cognitive and motor development is large in infants and toddlers. Given the previous results it is unknown at what age level children are capable of maintaining orientation during simulated self-movement while tracking of the correct target is prevented.

### The present study

To increase our understanding of the individual development of spatial cue use for orientation, in the present study we used a novel on-screen VR hide-and-seek paradigm mimicking movement of the participant within the environment. Specifically, we investigated whether two-and-a-half and near-three-year-olds are capable of maintaining orientation on the basis of visual cues alone, i.e., objects in the environment and visual optic flow, in the absence of physical movement and geometry information known to be helpful for children this age. Thirty-month and 35-month-olds performed a touch response task searching for an animated target in the presence of visual self-movement cues and landmark information. Crucially, within this paradigm, we prevented children from tracking the target's hiding position during the spatial transformation, thereby testing for orientation abilities. Selection of the oldest age group was determined by the previously described findings in the literature which indicate that the age range between 2 and 3 years is an important stage for development of spatial cue use (Bremner et al., 1994; Hermer and Spelke, 1994, 1996; Nardini et al., 2006). Based on these findings, we hypothesized that 35-month-old children would be better at maintaining orientation than a younger age group. For practical purposes we tested 30-month-olds as the youngest age group since children under the age of 2.5 years were expected to have difficulty understanding task instructions.

In addition to investigating group differences, a first attempt was made at linking developing behavioral capacities to visual spatial cue use for orientation in toddlers. Since little is known about individual differences in spatial cue use for orientation in young children, we made use of an easy to administer parental questionnaire that covers multiple domains of adaptive functioning. Using the Vineland Screener (Vineland-S; Scholte et al., 2008), we assessed children's level of adaptive functioning across the following four domains: communication, daily living skills, socialization, and motor skills to test for correlations between spatial task performance and these four domains as a measure of individual differences in the successful use of visual spatial cues for maintaining orientation. Given the small number of previous findings of individual differences in spatial cue use related to language or spatial action as measured by children's motor development and the child's opportunities to explore its surroundings (Hermer-Vazquez et al., 1999; Newcombe and Ratliff, 2007; Pyers et al., 2010; Shusterman et al., 2011; Newcombe et al., 2013), we hypothesized that rather than chronological age, individual development, as measured by scores on the subscales measuring language, motor and/or daily living skills measuring relative independence, were possible predictors for spatial task performance.

## Materials and methods

### Participants

Forty-five children took part in the experiment, 23 aged 30 months (±14 days; 12 male) and the remaining 22 aged 35 months (±14 days; 12 male). A further 3 children were tested, but were excluded from analysis: one 30-month-old for failing to finish the experiment, one 30-month-old due to a technical problem with video recording and one 35-month-old due to missing values. Children were recruited through a database held at the Baby Research Centre (BRC) in Nijmegen, the Netherlands. Written informed consent was obtained from the parents for each child according to a protocol approved by the local Radboud University Nijmegen Ethics Committee for Behavioral Research (ECG 27012011). Parents could choose between a children's book and 10 Euros for their child's participation. Parental report indicated that all children were typically developing, without significant birth histories.

### Stimuli

**Figure 1:**
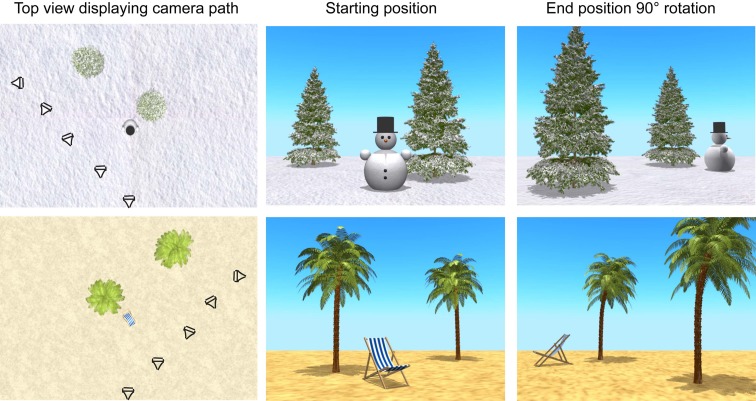
**Two virtual environments**. Top view of two environments displaying camera paths with camera positions and angles at five time points, and screen shots of starting and end position of camera, resulting in either a 90° turn to the left (in snow environment) or the right (in beach environment) of the center of the environment.

Experimental stimuli consisted of 16 movies created with open source animation suite Blender (www.blender.org). The movies featured a target, an animated bird, appearing in front of the camera, turning around and flying into one of two identical trees positioned at different distances within an open 3D environment. Once the bird had flown into one of the two trees, the camera perspective followed a path, mimicking self-motion, resulting in a perspective change 90° to the left or the right of the center of the environment, meanwhile preventing tracking of the bird's hiding position due to eye fixation on the correct tree. Figure 1 gives an overview of two environments with the camera paths and two screen shots for each environment. The camera was placed at the height of 0.9 Blender units, corresponding to a virtual height of 90 cm, i.e., at the average eye level of 2.5-3-year-olds. The camera was facing the center of the environment at a viewing angle of 5° upwards. The distance to the center of the environment (before and after the spatial transformation) was 6 meters, and the distance of the spatial transformation was 8.5 meters. The total duration of the turn was 4 s. To prevent tracking of the bird's hiding position due to eye fixation on the correct tree, while moving, the camera gradually turned toward the end position of the camera path, resulting in all objects in the environment temporarily disappearing from sight, only to face the center of the environment (and all objects) again when reaching the end of the path (see Online Movie for four examples of experimental trials).

**Figure 2:**
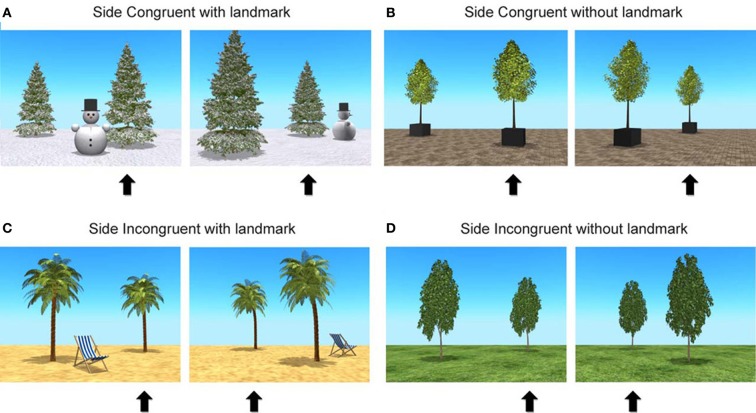
**Stills of stimulus movies**. Four different environments, in half of the trials containing a unique object to serve as landmark, with screen shots before and after a camera view change for Side Congruent (SCon) and Side Incongruent (SInc) trials, marking the hiding tree with a black arrow below the stills for illustration purposes. For simplicity, only movie stills with camera view changes 90° to the left of the center of the environment are presented. The figure shows **(A)** a SCon trial with landmark, and **(B)** a SCon trial without landmark, where the position of the hiding tree which was right before rotation remained the right tree after 90° rotation to the left, **(C)** a SInc trial with landmark, and **(D)** a SInc trial without landmark, where the position of the hiding tree, which was right before rotation, becomes the left tree after 90° rotation to the left.

Four 3D environments were used; a beach, an open square, a snow, and a park landscape. To assess subject-to-object relation use, in half of the trials the correct hiding place corresponded to the position of the tree relative to the child's body before the camera angle change, referred to as Side-congruent trials (SCon). In the other half, the trials were labeled as Side Incongruent (SInc), with the hiding place not matching the position of the tree relative to the child's body before the camera angle change (see Online Movie). In addition, to assess object-to-object relation use, in half of the trials a unique object was placed in the VR environment to serve as a landmark which could be used for reorientation (a beach chair, a wooden bench, a snowman, and a rabbit spring rider, respectively). This resulted in a total of 16 trials presented across four experimental conditions: Side-congruent with landmark (SCon+Lm), Side-congruent without landmark (SCon−Lm), Side-Incongruent with landmark (SInc+Lm), and Side-Incongruent without landmark (SInc−Lm). Figure 2 illustrates before and after screen shots of example trials across the four conditions. Position of the hiding tree (left/right and front/back), landmark (presence/absence), position of landmark (close to or further removed from hiding tree), and turn (90° left/right) were all fully counterbalanced.

### Procedure

Children were seated in front of a Hewlett-Packard 23 inch LCD touch screen monitor, positioned within arm's reach of the child. On top of the monitor an LG web cam was fixed that provided an image of the child's face and eye movements. The experiment was videotaped for the purpose of off-line assessment of children's attention to the movies. Stimuli were presented using a Dell laptop running Presentation software (Neurobehavioral Systems, Inc.).

Participants were told that they were about to play a game on the touch monitor. They were asked to carefully watch Pico, the purple bird, flying away and hiding somewhere. Upon hiding, the camera angle changed and afterwards the child was required to indicate where the bird was hiding by touching its hiding location on the touch monitor. X and y coordinates of the touch were recorded for analysis of correct and incorrect responses. Upon touching the screen, the bird reappeared from the correct tree, giving the child feedback on where it had been hiding. As a form of reward, the bird made a whistling sound when flying toward the camera whenever the child had touched the correct tree (see Online Movie for responses of three 30-month-old participants). Children were given one practice trial before starting. The presence of landmarks was manipulated between blocks of four trials, starting with a block with all four scenes containing a landmark. At least 2 different scenes were presented in between two identical scenes (e.g., square-*snow*-beach-park-*snow*) to avoid perseveration errors translating across identical environments possibly affecting these age groups (e.g., Deloache and Brown, 1983; Spencer et al., 2001). All throughout the experiment one of the parents was seated some distance behind the child. The experimenter was seated next to the child and ascertained whether the child wanted to proceed before starting the next trial. The total duration of the experiment was 15–20 min.

### Parental questionnaire

In the interest of exploring individual differences in task performance, children's level of adaptive functioning was assessed with the Dutch Vineland Screener 0–6 (Vineland-S; Scholte et al., 2008), a 72-item shortened version of the parent-report Vineland Adaptive Behavior Scales (VABS; Sparrow et al., 1984), originally developed for use in the evaluation of mental retardation. The Dutch Vineland-S is derived from the American VABS screener version and can be used for both individual diagnostic purposes and research assessing the adaptive behavior of a range of normally developing individuals (see also Van Duijn et al., 2009). For 38 children, information was gathered from one of the parents on everyday behaviors of the child across four domains: communication skills (19 items), daily living skills (16 items), socialization skills (19 items), and motor skills (18 items). The communication domain evaluates the receptive, and expressive language skills of the child, the daily living skills domain measures the child's independence by way of self-care activities as well as domestic and community interaction skills, the socialization domain covers interpersonal relationships, play and leisure time, and the motor skills domain measures both gross and fine coordination skills. Parents indicated on a three-point scale (0—no/never, 1—sometimes/partially, 2—yes/usually, or UN—unknown) whether the child exhibited the particular behavior in everyday life. The Vineland-S provides age-equivalent scores and standard scores for each domain, and a composite age-equivalent score for each child (Sparrow et al., 1993). Good reliability and validity of the Vineland-S have been established in a normal population (Evers et al., 2000).

### Touch response analysis

Each trial was coded as correct when children attended the movie and touched the correct tree on the touch monitor, and incorrect when children attended the movies, but touched the incorrect tree. In only very few instances children touched the landmark, sky or ground. These trials were also coded as incorrect. Trials where children did not attend to the video at one or both of the critical fragments of the bird flying toward one of the trees and hiding and the camera turning (as assessed on-line by the experimenter and in case of doubt off-line based on video recordings), were discarded from the analysis (on average, 10.9% in 30-month-olds and 12.5% in 35-month-olds). Next, mean accuracy scores were computed per child per condition and repeated measures ANOVAs and *t*-tests were performed. Significant interactions involving the factor Group were followed by separate within-group ANOVAs.

## Results

### ANOVA

**Figure 3:**
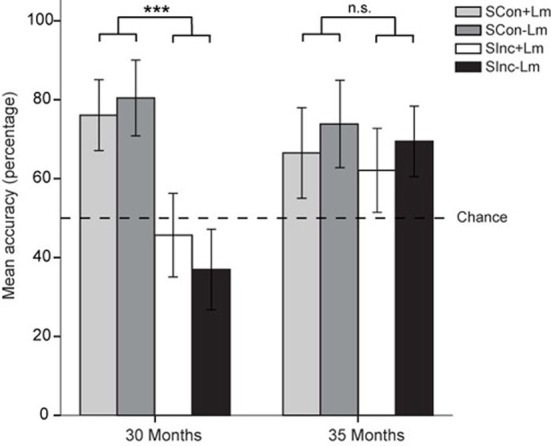
**Spatial orientation task performance**. Mean accuracy scores and 95% confidence intervals by age group for Side-congruent with landmark (SCon+Lm; light gray), Side-congruent without landmark (SCon−Lm; dark gray), Side-incongruent with landmark (SInc+Lm; white), and Side-incongruent without landmark (SInc−Lm; black) conditions. ^***^p < 0.001.

Mean accuracy scores by age group and experimental condition and 95% confidence intervals adjusted for repeated measures (Loftus and Masson, 1994) are displayed in Figure 3. Overall, both the 30-month and 35-month-olds performed above chance (50%) on the task with a mean performance accuracy of 59.8% for the 30-month-olds, *t*_(22)_ = 3.34, *p* = 0.003, *d* = 1.42, and 69.9% for the 35-month-olds, *t*_(21)_ = 5.78, *p* < 0.001, *d* = 2.52, indicating that both age groups understood the task and were not touching the screen at random.

A repeated-measures ANOVA with group (30 vs. 35 months) as between-subject factor and congruency (SCon vs. SInc) and landmark (present vs. absent) as within-subject factors revealed significant main effects of group, *F*_(1, 42)_ = 4.99, *p* = 0.031, η^2^_p_ = 0.11, and of congruency *F*_(1, 42)_ = 24.84, *p* < 0.001, η^2^_p_ = 0.37, and a significant group x congruency interaction, *F*_(1, 42)_ = 12.54, *p* = 0.001, η^2^_p_ = 0.23, reflecting a developmental change in profile across congruency conditions. Overall, no main effect of landmark was found, *F*_(1, 42)_ = 1.99, *p* = 0.166, η^2^_p_ = 0.05, nor an interaction of landmark x congruency, *F*_(1, 42)_ < 1, η^2^_p_ = 0.01. However, a group x landmark interaction indicated that landmark use also differed between the two age groups, *F*_(1, 42)_ = 4.22, *p* = 0.046, η^2^_p_ = 0.09.

A follow-up within-group ANOVA for the younger age group revealed a significant main effect for congruency, with higher accuracy scores in the side congruent compared to the SInc conditions, *F*_(1, 22)_ = 29.03, *p* < 0.001, η^2^_p_ = 0.57. No significant effect of landmark, *F*_(1, 22)_ < 1, η^2^_p_ = 0.11, nor a significant interaction of congruency x landmark, *F*_(1, 22)_ = 29.03, *p* < 0.001, η^2^_p_ = 0.13 was found. For the older age group no difference in performance on congruency conditions was found, *F*_(1, 20)_ = 1.52, *p* = 0.232, η^2^_p_ = 0.07. However, a significant main effect of landmark indicated that the 35-month-olds performed worse on trials with a landmark present, compared to trials without a landmark present *F*_(1, 20)_ = 5.21, *p* = 0.034, η^2^_p_ = 0.21. Here too, no interaction of congruency x landmark was found, *F*_(1, 20)_ < 1, η^2^_p_ = 0.01.

Collapsed across the landmark manipulation, SCon trials were above chance for both age groups, *t*_(22)_ = 8.37, *p* < 0.001, *d* = 3.57 for 30-month-olds, and *t*_(21)_ = 8.26, *p* < 0.001, *d* = 3.6 for 35-month-olds (Figure 3). However, relative to 30-month-olds an increase in correct responses on the SInc trials in the older children is present, with performance significantly below chance for the 30-month-olds, *t*_(22)_ = −2.82, *p* = 0.01, *d* = −1.21, to significantly above chance for 35-month-olds, *t*_(21)_ = 4.97, *p* < 0.001, *d* = 2.17.

Based on the observation that during testing children referred to the snowman and rabbit more often than the beach chair and bench, we decided to investigate the absence of a positive effect of landmark cues further. In total, 20 children (fifteen 30-month-olds and five 35-month-olds) made spontaneous references to the landmark in the environments, with some naming multiple landmarks during the experiment; 12 children referred to the snowman (with a total of 18 references), 8 to the rabbit spring rider (9 references), 5 to the beach chair (7 references) and 3 to the bench (3 references).

To this end, we subdivided the landmarks used in the study into landmarks that potentially would be of interest to the children (i.e., a snow man in the snow scene and a rabbit spring rider in the park scene, together accounting for 73% of the references) and landmarks that would be of less interest (a beach chair in the beach scene and a bench in the square scene, i.e., 27% of the references).

**Figure 4:**
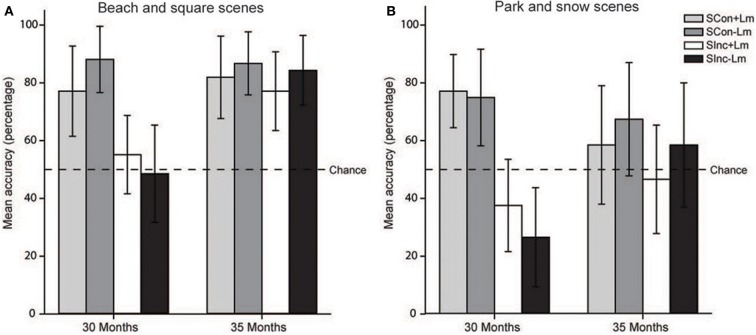
**Spatial orientation task performance after split on scene type**. Mean accuracy scores and 95% confidence intervals by age group and condition after split on type of scene, which either (had) contained **(A)** a landmark of no particular interest to children (beach chair/wooden bench) or **(B)** a landmark of much interest to children (snow man/rabbit spring rider).

Figure 4 displays the mean accuracy scores and 95% confidence intervals after a split on scene type. The interaction between congruency and group was not influenced by scene type. Instead, overall performance drops when a VR environment was presented that in the first trial (had) contained a landmark that would be of particular interest to young children, relative to VR environments that (had) contained a “less interesting” landmark. The confidence intervals in Figure 4 indicate that for the older age group, relative to the “uninteresting landmark scenes”, in the “interesting landmarks scenes” performance drops from above chance to chance level. For the 30-month-olds, the main effect of congruency persists in the “interesting landmark scenes”, with performance on SCon trials remaining constant at above chance level and performance on SInc trials dropping from chance level to significantly below chance level.

### Multiple regression analyses

**Figure 5:**
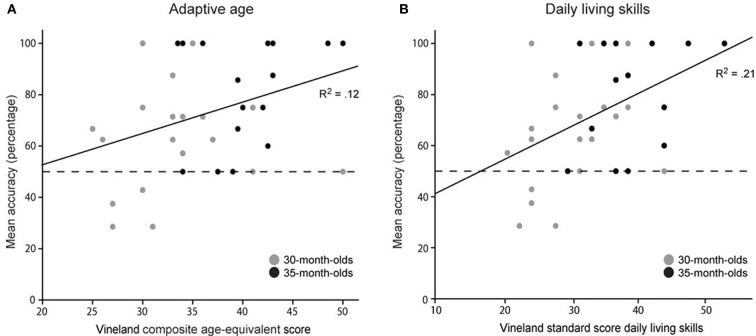
**Individual spatial task performance on non-distracting trials (i.e., beach and square environments)**. Scatter plots indicating linear correlations between **(A)** Vineland composite age-equivalent score and mean accuracy, and **(B)** Vineland standard score daily living skills and mean accuracy.

Given the apparent effect of scene type, we decided to perform two regression analyses, one with performance on all trials as the dependent measure and a second regression analysis including the beach and square environments only. Table 1 reports descriptive statistics of task performance, age, sex and the adaptive age measures. In order to assess the relation between adaptive behavior and task performance we first examined positive correlations between subjects' Vineland composite age-equivalent score and mean accuracy scores for all trials, and non-interesting landmark scene types, separately. Whereas the correlation based on all trials showed a trend toward significance, *r*_(36)_ = 0.25, *p* = 0.065, the correlation of adaptive age with accuracy on beach and square trials reached significance, *r*_(36)_ = 0.35, *p* = 0.016 (see Figure 5A). Relationships between spatial skills, age, gender, and behavioral domains from the Vineland-S were assessed by means of multiple regression analyses (forward and backward) with mean accuracy scores on either all trials, or on beach and square trials as the dependent measure and gender and chronological age and the four subscales of the Vineland-S as independent measures. Collinearity statistics indicated no problems of multicollinearity with these predictors (all tolerance levels above 0.47). Both models reached significance with the Vineland subscale daily living skills as the sole significant predictor for spatial task performance: β = 0.37, *F*_(1, 36)_ = 5.57, *p* = 0.024, *R*^2^ = 0.13 for the model with mean accuracy scores based on all trials as the dependent measure, and β = 0.46, *F*_(1, 36)_ = 9.78, *p* = 0.003, *R*^2^ = 0.21 (see Figure 5B) for the model based on the beach and square trials. These findings suggest that the individual adaptive behavior component reflecting children's relative independence may have an influence on spatial task performance.

**Table 1:** **Descriptive statistics for all measures**.

	***N***	**Mean**	***SD***	**Minimum**	**Maximum**
Accuracy all scenes	38 (45)	0.64 (0.65)	0.16	0.31	0.93
Accuracy beach + square	38 (45)	0.73 (0.74)	0.22	0.29	1.00
Age	38 (45)	32.2	2.5	30	35
Adaptive age	38	36.8	6.4	25	50
Communication	38	38.7	7.2	24	51
Socialization	38	37.2	9.3	23	55
Daily living	38	34.7	10.9	21	53
Motor	38	36.7	6.3	25	50

## Discussion

The current study investigated the ability of 2.5–3-year-olds to maintain orientation in an on-screen VR task, and assessed factors that may influence spatial task performance. Thirty-month and 35-month-olds performed a touch response task searching for an animated target in the presence of visual self-movement cues and landmark information. Above chance performance on all conditions indicated that children aged 35 months were capable of using selective visual cues to compensate for the spatial transformation (Figure 4). By contrast, performance of the 30-month-olds did not exceed or was even significantly below chance level in the SInc conditions, indicating that they failed at optimally using the visual spatial cues present in the VR environment. Note that overall performance in both age groups exceeded chance level, indicating that they understood the task and were not touching the hiding places on the screen at random. Failure of the younger age group to locate the target in the SInc, therefore, cannot be ascribed to an inability to understand the task instructions. Instead, these results reveal a developmental change in spatial task performance in 2-year-olds within a few months only, where children learn to use selective visual information for successful orientation. Correlational analyses revealed that rather than chronological age *per se*, individual differences may play a role in successful use of visual cues for spatial updating in an orientation task.

### Viewpoint independent processing or spatial updating?

In our paradigm, orientation via viewpoint independent processing should have benefited from the presence of landmarks in the environment, as viewpoint independent processing solely depends on the information of intra-object locations to encode the topological structure of the environment. On the basis of two identical objects in an otherwise empty environment it is not possible to unambiguously predict one's location from a novel vantage point on the basis of viewpoint independent processing alone (e.g., instead of a rotation to the left, a 90° rotation to the right in the without landmark trials would have resulted in identical screen shots after camera turn in Figures 2B,D). Adding a third and unique object in the landmark conditions (Figures 2A,C) would allow for a response based on viewpoint independent processing. The changing camera angle would have provided no additional useful information to the children other then the introduction of a viewpoint shift.

Above chance performance in the absence of a positive landmark effect in the 35-month-olds suggests that overall the older children did not need to rely on the presence of a unique landmark in the environment to find the hidden target. Therefore, rather than solving the task based on viewpoint independent processing, optic flow cues provided by the camera path were used for spatial updating processes in the 35 month-olds, providing additional evidence that in cases of a simple rotation and translation, optic flow cues can be sufficient for reorientation. By contrast, the younger age group failed at retrieving the hidden target in the SInc conditions. This indicates that they were incapable of using the visual information (both path integration and landmark information) in the VR environment for successful orientation. Above chance performance on the SCon and below chance performance in the SInc conditions reveal that the 30-month-olds followed a response pattern opting for the spatial position of the tree before the viewpoint manipulation. It cannot be concluded if this response pattern is due to an egocentric frame of reference (i.e., left/right position of the tree relative to the body) or if the response is made in relation to other cues like the computer screen or the experimental room.

### Strategy use

Interestingly, when failing to use the available visual information, younger children made use of a spatial strategy related to left and right hand side, rather than an object-based spatial strategy. Although matched for size in 3D, the objective size of the two trees on the screen differs (see Figure 2). If the 30-month-olds were unaware of the viewpoint manipulation in our virtual environments, they also may have opted for a strategy based on disambiguating object processing related to the subjective tree size difference. Studies show that infants as young as 4.5 months of age are capable of disambiguating objects on the basis of their size (Wilcox and Baillargeon, 1998a,b; Wilcox, 1999). In our paradigm, the use of this particular strategy would have resulted in a reversed response pattern with above chance performance on the SInc condition and below chance in the SCon conditions. This finding suggests that 30-month-olds were aware of our viewpoint manipulation in a 3D environment, but were unable to successfully use the visual cues for maintaining orientation.

These results are reminiscent of early perspective taking studies where children were asked to anticipate location relative to different potential vantage points by imagining the perspective of another person (see Newcombe, 1989, for a review). In most of these studies, children were presented with pictures or models in which represented objects did not correspond to the perceptually present frame of reference of the child. Children aged 3.5–6 years old have been shown to be extremely poor at tasks involving such a conflict between the actual and imagined frames of reference (Rieser et al., 1994), and errors made by these children were primarily “egocentric” (i.e., with respect to the actual body frame of reference). Alternatively, by using questions about which object would occupy a specified position with respect to another observer, Newcombe and Huttenlocher (1992) found that children as young as 3 years understand that other vantage points command different views, and that they should not code location only with respect to their own body position. In the present study, even though we did not ask children to imagine another point of view, they were presented with a vantage point shift from before and after the 90° rotation. This may have led to a conflict between these two frames of reference, resulting in an “egocentric” response pattern in the younger age group similar to the early perspective taking studies. Moreover, studies have shown that in both children and adults, perspective taking improves after self-movement, even in the absence of vision (Rider and Rieser, 1988; Rieser and Rider, 1991). The optic flow cues used for spatial updating may have aided the 35-month-olds in the present study to overcome this conflict between the two reference frames.

### Absence of landmark use

Even though during testing, almost half of the children spontaneously referred to the unique objects in the VR environments, the 2.5–3-year-olds in our study did not benefit from these additional objects present in the environments. Whereas 30-month-olds showed a similar performance when landmarks were present or absent, the 35-month-olds even performed worse on environments containing a landmark. This appears to be in contrast to findings in the literature on landmark use in spatial reorientation in young children, (Newcombe et al., 1998, 2010; Learmonth et al., 2001; Smith et al., 2008). There are a few possible explanations for the lack of a positive landmark effect in our study.

A first explanation is that children under the age of 3 years were incapable of using the landmarks, either because they were too far removed from the target location, or because they were viewed from a large angle difference. Studies on children's ability to use landmarks in reorientation paradigms have shown that a distinction must be made between objects that directly mark a goal location (so-called beacons) vs. objects that serve as associative cues to mark a goal location (Lee et al., 2006; Lew et al., 2006; Lee and Spelke, 2010b). These studies revealed that preschool children are able to use beacons to find a target location, but fail to use freestanding objects further removed from the target location as reliable cues for reorientation. It is conceivable that children would have been able to use the landmark in locating the target if we had included a condition where our target hid behind the unique object within the environment or a tree adjacent to the landmark, and the object served as a direct marker of the target location. Using a unique object as an indirect marker for the goal location may have been too difficult for this age group. Additionally, a study by Nardini et al. (2009) showed that children younger than 5 years of age were incapable of using landmarks from an opposite viewpoint unless movement information about their displacement was available. It was argued that previous findings of landmark use in relocating hidden objects from novel viewpoints may be supported by effective view matching processes, rather than by the encoding of the spatial environment (but see Lee and Spelke, 2011, for a different view). Possibly, our 90° displacement is too large to allow for view matching processes and children may have been too young to effectively use landmarks from a novel viewpoint. Especially in the younger age group, where children were incapable of using the optic flow cues for spatial updating.

However, an alternative explanation for the lack of a positive landmark effect is not related to the age of our participants but follows from the adaptive combination view, which predicts that reliance on featural information will increase as landmarks are more distal, larger, and invariantly present (Newcombe and Ratliff, 2007). In an attempt to fully counterbalance our design we used identical environments where landmarks were not always present. As landmarks can be expected to be less relied upon when their presence is unstable, we may inadvertently have introduced a lack of certainty in encoding of landmark information, resulting in the absence of a landmark effect.

Even though these options may account for the lack of a positive landmark effect, they cannot account for the reversed landmark effect in the 35-month-olds. In investigating the reversed effect further an analysis of the four different VR environments used in the study revealed performance pattern differences across the four environments. Overall performance dropped when a VR environment was presented that contained or in the first trial had contained a landmark that is assumed to be of interest to the children, relative to VR environments that (had) contained a “less interesting” landmark (Figure 3). We propose that this larger interest in the landmarks can either be due to the toy character and/or to the landmarks being potentially animate/mobile agents. Therefore, the inferior performance on landmark trials in 35-month-olds is possibly the result of a conflict between attention to the location of the (previously present) interesting object and the spatial encoding of the bird's hiding place. However, the “uninteresting” landmarks still did not facilitate performance revealing that the near-3-year-olds are able to rely on optic flow cues only and either could not or simply did not need to use the unique objects in the VR environment. Future studies investigating landmark use in spatial orientation and navigation in young children should consider that using unique objects that are of specific interest to children (e.g., toys) could potentially distract children, and may cause attention to be directed away from the task.

Crucially, however, when distracted by the interesting landmark scenes, the 30-month-olds still showed an enhanced preference for “egocentric” responding (i.e., chose the position of the tree that corresponded closest to its position on the screen before the turn), whereas the 35-month-olds now performed at chance level instead. This “egocentric” response bias in the younger children is abandoned when children are successful in using the optic flow for path integration, but cannot recall where the target is hiding. In those cases children start to show a guessing pattern. Therefore, in the context of identical cues available, within 5 months, we see a developmental change in the search strategy when faced with uncertainty about a target location. Taken together, these results suggest that whereas the 35-month-olds are capable of using optic flow cues for reorientation, the 30-month-old children possibly need more cues for successful spatial updating, such as actual motion (providing additional vestibular and proprioceptive cues).

### Individual differences

So far, we have discussed results at the group level, speaking of 30- and 35-month-olds as separate groups. However, this study shows that the use of particular spatial cues to arrive at a spatial representation of the environment changes over time, but more importantly, that this process differs between individuals. Our results indicate that rather than chronological age *per se*, individual development may determine success in spatial cue use for maintaining orientation. It has been previously hypothesized by others that individual differences in children's orientation abilities may be affected by factors such as language or spatial action as measured by children's motor development and the child's opportunities to explore its surroundings (Hermer-Vazquez et al., 1999; Newcombe and Ratliff, 2007; Pyers et al., 2010; Shusterman et al., 2011; Newcombe et al., 2013). In the present study, correlations with spatial task performance were observed for adaptive behavior measures, which were not driven by the child's motor or communication skills (including a measure for language development), but by the child's daily living skills (Figure 5). This finding indicates that children who are relatively more independent (e.g., were able to dress themselves, were toilet-trained, and/or were more aware of danger in and around the house) in comparison to their peers, were more successful in identifying the correct target location after reorientation. A tentative explanation could be that, children who possess more personal independence skills, in everyday life are given more opportunities for spatial exploration relative to their peers who are more dependent on their care-takers. These greater opportunities for exploration may allow them to be more aware of their spatial surroundings, and as a result these children performed better on our spatial reorientation task. Preliminary support for this hypothesis follows from a recent longitudinal study into the relation between the degree of spatial exploration during infancy, as based on retrospective parental reports, and spatial memory at ages 4 and 6 years (Oudgenoeg-Paz et al., 2013). Results showed that spatial exploration (but not age of attainment of self-locomotion) indeed positively predicted spatial memory at both ages, indicating that spatial exploration predicts spatial memory even over longer periods of time. Future research will have to explore this specific hypothesis further.

## Conclusion

These results are consistent with previous research showing that during early childhood there is a gradual improvement in the use of various spatial cues. Moreover, given identical cues, a developmental transition in spatial cue use can be witnessed within a relatively short period of 5 months. Also, this study shows that rather than chronological age, individual development may determine success in using visual cues for spatial updating. Future research will have to assess whether individual differences at this young age persist, and if so, what mechanisms underlie these individual differences, and whether they translate onto adult differences in spatial cue use or strategies used in navigation.

## Author contributions

Danielle van den Brink and Gabriele Janzen designed the experiment. Danielle van den Brink performed the experiment and analyzed data. Danielle van den Brink and Gabriele Janzen wrote the paper.

### Conflict of interest statement

The authors declare that the research was conducted in the absence of any commercial or financial relationships that could be construed as a potential conflict of interest.

## Supplementary material

Online Movie **Video showing four experimental trials and responses of three 30-month-old children**.
